# Comparative analysis of the hemostatic, analgesic and healing effects of cyanoacrylate on free gingival graft surgical wounds in donor and recipient areas: a systematic review

**DOI:** 10.1007/s10856-021-06573-z

**Published:** 2021-08-18

**Authors:** Aretha Heitor Veríssimo, Anne Kaline Claudino Ribeiro, Ana Rafaela Luz de Aquino Martins, Bruno Cesar de Vasconcelos Gurgel, Ruthineia Diógenes Alves Uchoa Lins

**Affiliations:** Department of Dentistry, Federal University of Rio Grande do Norte (UFRN), Av. Salgado Filho, 1787, Lagoa Nova, Natal, 59056-000 Brazil

## Abstract

**Abstract:**

To analyze the hemostatic, Dsurgical wounds in donor and recipient areas of free gingival grafts (FGG). Five databases (PubMed, Scopus, Science Direct, Cochrane and Web of Science) were searched up to March 2021 (PROSPERO CRD42019134497). The focus of the study (cyanoacrylate) was combined with the condition (periodontal surgery OR free gingival graft OR free soft tissue graft OR autografts), and outcome (healing OR epithelialization OR pain OR analgesia OR bleeding OR hemostasis OR hemostatic). Studies reporting cyanoacrylate isolated or associated with another substance in FGG stabilization and closure were investigated and assessed for the quality and risk of bias through the Cochrane Manual. Six studies with 323 participants were included. Evaluation of the quality and risk of bias highlighted a low risk for four articles, intermediate for one and unclear for another. The use of cyanoacrylate associated or not with the hemostatic sponge or the platelet-rich fibrin was more effective in healing (three studies), analgesia (four studies), and hemostasis in one study (*p* < 0.05). However, groups with the association in cyanoacrylate showed superior healing, and analgesic action to the isolated cyanoacrylate group. In addition, two studies demonstrated that cyanoacrylate use reduces surgery duration, one study showed that it reduces postoperative sensibility, and another present hemostatic effect (*p* < 0.05). There is scarce literature for the use of cyanoacrylate in FGG wounds indicates that it can promote a minor inflammatory response, reduce operation time, does not interfere with healing, relieves postoperative discomfort, and suggests the possibility immediate hemostasis. Its use presents an alternative to suturing in FGG surgeries. But, the limited number of cases and the relative heterogeneity of the included studies suggest caution in generalizing the indication.

**Clinical relevance:**

Cyanoacrylate seems to present analgesic effects and less pain when applied to wound closure and covering donor and recipient areas reducing the need for postoperative analgesic medication; and has a healing effect in the closure of the donor area on the palate. In addition, it can reduce bleeding time after surgery, and prevents late bleeding during the first postsurgical week. **Scientific justification:** To evaluate the hemostatic, analgesic and healing actions of cyanoacrylate compared to the suture thread and other agents when used to close surgical wounds from periodontal free gingival graft surgical wounds in both the donor and recipient areas of the graft. **Main findings:** The use of cyanoacrylate individually or in association with wound dressing agents presents analgesic effects because the patient reports less pain experienced when cyanoacrylate is applied to the wound closure and covering, thereby reducing the need for postoperative analgesic medication. In addition, a healing effect is observed in the closure of the donor area on the palate; as well as it seems to present hemostatic effects, reducing the bleeding time after surgery, and preventing late bleeding during the first postsurgical week. **Practical implications:** Dentists may cautiously apply cyanoacrylate after periodontal surgeries for free gingival graft in both the donor and recipient areas of the graft. However, they must consider the limitations of the surgery, tension-free positioning, the patient’s dyscrasia and postoperative care, constituting a set of predictors for adequate clinical decision-making. Widespread use of such material for all patients and surgical configurations may not be recommended.

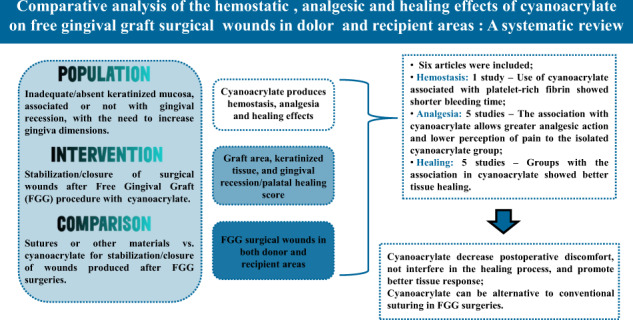

## Introduction

The choice of material to establish good tissue synthesis is extremely important for postoperative success. Tissue synthesis aims to maintain the tissues well coaptated in order to accelerate the healing process, prevent bleeding and contribute to forming and maintaining the blood clot, thereby avoiding infection at the site, contamination of the surgical wound and postoperative pain [1, 2].

Cyanoacrylate adhesives have been extensively used for closing skin wounds and in several surgical procedures involving skin, mucous membranes and different tissues, including oral tissues [3–5]. These adhesives have been applied in the oral cavity for flap closure, gingival graft fixation, and pulp capping [1, 6–8]. With the emergence of such chemical adhesives and due to the interference of conventional sutures in the tissue healing process, some professionals began to replace sutures with tissue adhesives, especially cyanoacrylates, being commonly used in the synthesis of surgical wounds [3].

The main advantages of bioadhesive materials are generally attributed to the following factors: high tissue compatibility and long half-life; presence of hemostatic, analgesic and antibacterial properties; high potential for adhesiveness and biodegradation; the ability to maintain the position/stabilization of injured tissues; and to trigger a mild inflammatory response [3, 4, 6, 9].

In periodontal surgeries, when the free gingival graft is used to increase the range of keratinized mucosa, the donor tissue thickness and the graft stabilization in the recipient area are vital to protect local vessels against damage and dehydration, thereby decreasing the possibility of bleeding, tissue retraction, and consequently contamination of the surgical wound and postoperative pain [2]. It is possible that techniques which promote surgical wound closure without sutures, for example, tissue bioadhesives, may have a hemostatic effect and still decrease or even prevent tissue retraction and its negative consequences [10].

In this context, the objective of this systematic review was to analyze the hemostatic, analgesic and healing effects of cyanoacrylate when applied to surgical wounds in donor and recipient areas of free gingival grafts (FGG), and compare them with those produced using conventional sutures and other materials and techniques.

## Material and methods

This systematic review followed the statement of the Preferred Reporting of Systematic Reviews and Meta-analysis (PRISMA) and checklist [11]. The protocol was registered in PROSPERO (International Prospective Register of Systematic Reviews), at the UK National Institute for Health Research, University of York, Center for Reviews and Dissemination, under code CRD42019134497 (https://www.crd.york.ac.uk/PROSPERO/display_record.php? RecordID=134497). This systematic review addressed a clearly focused issue, adopting the population, intervention, comparison and outcome (PICO) method [12].

### Focused question

What is the difference in the hemostatic, analgesic, and healing effects of cyanoacrylate compared with sutures or other materials when applied after the surgical procedure of free gingival graft surgical wounds in donor and recipient areas?

This question considered the following PICO definitions:

#### Population

Patients with inadequate, insufficient or absent keratinized mucosa, associated or not with gingival recession, with the need to increase their gingiva dimensions.

#### Intervention

Stabilization and closure of surgical wounds after FGG procedure with the use of Cyanoacrylate.

#### Comparison

Sutures or other materials vs cyanoacrylate for stabilization and closure of wounds produced after free gingival graft surgeries.

#### Outcome

Cyanoacrylate produces hemostasis (immediate hemostasis after surgery measured in minutes, and bleeding during the first week after surgery), analgesia (pain scores in the postoperative visual analog scale-VAS and consumption of analgesics), and healing effects (dimensions measured (height and length), graft area, keratinized tissue, and gingival recession or palatal healing score in the postoperative VAS after the surgical procedure of free gingival graft in free gingival graft surgical wounds in both donor and recipient areas (Table 1).

**Table 1:** Inclusion/exclusion criteria for this study

Inclusion criteria	Exclusion criteria
Studies published in the English language	In vivo or in vitro studies
Controlled and RCT studies	Non-randomized studies
Studies carried out in patients undergone to the surgical procedure of FGG surgery	Prospective or retrospective case series, cohort and case-control studies
Studies with groups of at least ten participants	Studies with insufficient information on cyanoacrylate application after the surgical procedure of FGG
Human studies with inadequate, insufficient or absent keratinized mucosa, associated or not with GR, with the need to increase their gingiva dimensions	Studies which did not report cyanoacrylate action OR in the bleeding, OR in the experience of pain, OR in the healing of surgical wounds produced in donor or recipient areas of FGG
	Studies in which cyanoacrylate was not applied to FGG donor or recipient areas

*RCT* Randomized clinical trial studies, *GR* Gingival recession, *FGG* Free gingival graft

##### Information sources

Two authors (AV, AR) independently performed manual and electronic bibliographic searches in the following databases: PubMed, Scopus, Science Direct, Web of Science, and Cochrane including studies published until March 26, 2021. A third author (RL) was consulted when there were disagreements between the first two authors (AV, AR). Controlled terms (MeSH) and words were combined whenever possible. Terms not indexed in MeSH were also applied along with other filters, meaning that free terms were also used. An additional manual search through the bibliographic references of the studies included in this study was also carried out. Unpublished studies or gray literature were excluded, as they presented insufficient reports for our analysis.

The search strategy combined the search terms applied according to the focus of the study (cyanoacrylate), the condition (periodontal surgery OR free gingival graft OR free soft tissue graft OR autografts) and the outcome (healing OR epithelialization OR pain OR analgesia OR bleeding OR hemostasis OR hemostatic).

##### Literature selection and data extraction protocol

The screening of titles and abstracts was independently performed by two reviewers (AV, AR). Any disagreement was resolved through discussion or consultation with a third reviewer (RL). All articles considered potentially eligible were evaluated by reading the full text according to the inclusion and exclusion criteria in order to track all titles and determine their suitability for analysis. These criteria were decided by two consensus reviewers (AV, AR).

The data of interest, including the general description of the studies (title, authors, publication year, city, country, research period and study location), the characterization of the studied populations (number of patients, mean age and gender); indication for the free gingival graft (area/type of recession), the type of intervention (graft fixation or wound dressing), the sample unit (donor or recipient area of the free gingival graft), the study groups, outcome measures, clinical results (hemostatic, analgesics and healing), additional outcomes, follow-up time and conclusions were extracted from selected studies and organized in tables.

##### Risk of bias assessment

The quality assessment of the included studies was carried out according to the type of study used in the review, with only controlled and randomized clinical trials being selected. Thus, the referred studies were evaluated according to the RoB 1.0 tool in the Cochrane collaboration manual.

## Results

### Study selection and characterization

**Fig. 1:**
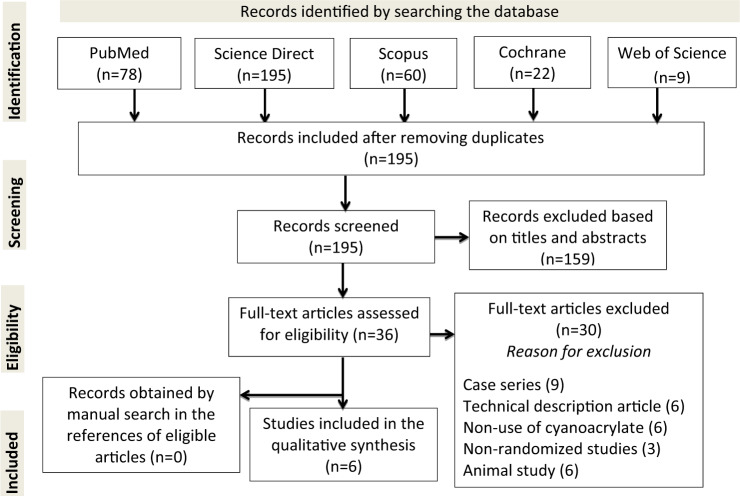
PRISMA flowchart of the study selection process. Records identified by searching the database

The systematic search of electronic databases identified 78 articles in PubMed, 195 in Science Direct, 60 in Scopus, 9 in Web of Science, and 22 in Cochrane. However, 195 articles remained after removing the duplicates and reading the titles, with 36 of them possibly eligible after review at the titles and abstracts level. Thus, six articles were included after reviewing the full texts. No articles were obtained by manually searching the references of eligible articles, and the reasons justifying the excluded studies are described in the PRISMA flowchart (Fig. 1). The included studies are presented in detail in Tables 2 and 3.

**Table 2:** Characteristics and description of included studies, sample (n), gender, mean age, indication for FGG (area/type of recession), type of intervention and follow-up

Authors (year)	Sample, *n*	Gender, (mean age, years)	Indication (Area/type of recession)	Sample unit (FGG)	Type of Intervention	Follow-up
Barbosa et al., 2009.	24	10 men, 14 women (37.6 years)	Inadequate or absence keratinized gingiva (Buccal sites of mandibular incisors/Miller’s class I–II recessions)	Receptor	Fixation of the FGG in the receptor bed.	0, 15, 30, 45 and 90 days
Gümüş and Budunel, 2014.	45	CoG: 1 men, 14 women (40.27 ± 4.83) CyG: 1 men, 14 women (37.60 ± 9.07) MG: 1 men, 14 women (34.80 ± 7.28)	To increase the keratinized gingiva width without any attempt of root closure. (One or two lower anterior teeth/Miller class III–IV recessions)	Receptor	Fixation of the FGG in the recipient bed.	0, 1, 3, 6 months
Ozcan et al., 2017.	125	NR, PRF:(34.55 ± 7.64) BC: (37.11 ± 4) WG: 37.61 ± 6.64)	Isolated gingival recession defects (Mandibular and maxillary anterior teeth)	Donor	Palatal wound healing after FGG harvesting.	Hemostatic and analgesic: 1–28 days Healing: 1–4 weeks.
Tavelli et al., 2018.	50	CG: 5 men, 5 women (46.4 ± 14.4) PaG: 3 men, 7 women (45.3 ± 11.5) SG: 6 men, 4 women (54.7 ± 10.3) PpG: 2 men, 8 women (52.8 ± 7.1) DLP: 1 men, 9 women (50.9 ± 11.5)	Single or multiple recession defect (with DGG) (Miller Class I, II or III). To increase keratinized tissue around implants (with FGG)	Donor	Hemostatic treatment after palatal gingival harvesting	1–14 days
Tavelli et al., 2019.	44	CG: 11 men, 11 women (52.6 ± 9.3) TG: 4 men, 18 women (50.86 ± 12.55)	Single or multiple recession defects (with DGG) (maxilla or mandible (canine to canine)/(Miller class I, II or III) in natural teeth or dental implants.	Donor	Palatal wound dressing after EGG harvesting	1–14 days.
Stavropoulou et al., 2019.	35	Suture group: 9 men, 9 women (58.5 ± 13.52) Cyanoacrylate group: 1 men, 16 women (53.18 ± 20.2)	Harvesting of CTG (palate).	Donor	Palatal wound dressing after FGG harvesting.	1–7 days

*FGG* Free Gingival Graft, *CoG* Conventional group, *CyG* cyanoacrylato group, *MG* Microsurgery group, *PRF* Platelet-rich fibrin, *BC* butyl-cyanoacrylate, *WG* wet gauze, *CG* Control group, *PaG* Periacryl group, *SG* Spongostan group, *PpG* Peripac group, *DLP* Double-Layered Protection - Spongostan + Periacryl group, *EGG* Epithelialized Gingival Graft, *CTG* Connective Tissue Grafts

**Table 3:** Summary of the analyzed studies

Authors (year)	Study groups	Evaluation Time (min)	Evaluated parameters	Hemostatic Outcome	Analgesic outcome	VAS score (days)	Healing outcome
Barbosa et al., 2009.	G1 - FGG + ethyl cyanoacrylate (12) G2 - FGG + sutures (12)	NR	Dimensions (height and length) of keratinized gingiva before and after surgery: recorded with a digital caliper after the use of Schiller’s solution.	NR	NR	NR	NS between the two groups. Both groups had similar dimensional changes in graft area.
Gümüş and Budunel, 2014.	CoG - FGG+ conventional procedures (15) CyG - FGG + cyanoacrylate (15) MG - FGG + microsurgery technique (15)	CoG: 37.33 ± 2.13 CyG: 26.87 ± 2.13 (p˂0.05) MG: 44.13 ± 3.46	Initial time of starting the incision and final time after graft stabilization Pain: Visual analogue scale (VAS) Clinical photographs of graft area, keratinized tissue, and gingival recession: recorded with a specific software (ImageJ).	NR	CyG: Pain in the recipient area was lower at all follow-up times (day 1–6) (*p* < 0.05)	Day 1–6: Decreased in all groups MG: lower than the other groups (*p* < 0.05).	CYG: graft retraction was lower (*p* < 0.05) Graft shrinkage (at 6 months): GYG: 18.53%; CoG: 32.50%; MG: 36.33%
Ozcan et al., 2017.	PRF group: PRF + BC adhesive (42) BC group: BC adhesive alone (42) WG group: sterile WG compression (41)	IBT time (*p* **=** 0.001): PRF: 0.57 ± 0.15) BC: 1.65 ± 0.69) WG: 3.18 ± 0.61) DB time (Day – %): 1- PRF: 0; BC: 81.9; WG: 90.2 (*p* = 0.001) 2 – PRF: 0; 0; WG: 82.9 (*p* = 0.001) 3 - PRF: 0; 0; 80.5 (*p* = .0001) 4 - PRF: 0; 0; 70.7 (*p* = .0001) 5 - PRF: 0; 0; 22.0 (*p* = .001) 6 - PRF: 0; 0; 9.8 (*p* = .038) 7 - PRF: 0; 0; 2.4 (*p* = .309)	Pain: Visual analogue scale (VAS) Immediate hemostasis after surgery (min), and bleeding during the first week after surgery Wound epithelialization parameter was assessed clinically and by means of color photographs The sensibility and sensory loss: assessed with a periodontal probe, rubbing movement, and pin pressure nociception.	PRF immediate hemostasis faster than the BC which was better than the WG (*p* < 0.05) PRF: no bleeding on the 7-day of follow-up BC: bleeding only on the 1st day in 34 patients WG: bleeding in the first week (*p* < 0.05)	PRF: lower pain experience than BC until the day 6 BC: pain experience lower than WG in the first 2 weeks All groups showed similar results after the 14th day (*p* < 0.05)	1. PRF:2.00; BC: 4.55; WG:6.10 2. PRF: 1.29; BC: 3.90;WG:5.22 3. PRF: 0.36; BC:1.90; WG:3.22 4.PRF: 0.12; BC: 1.21; WG:2.41 5. PR:0.02; BC:0.88; WG:1.98 6.PRF:0; BC:0.12; WG:1.29 7.PRF:0; BC: 0; WG:1.02 PRF and WG (*p* = 0.001); BC and WG (*p* < 0.05), except to 21 and 28 days PRF and BC (*p* < 0.05) up to day 5.	PRF and BC: better epithelialization compared to WG (*p* < 0.05) PRF was superior to BC only in the 2nd week.
Tavelli et al., 2018.	CG: control group with suture (10) PaG: cyanoacrylate bioadhesives (10) SG: hemostatic absorbable gelatin sponge (10) PpG: periodontal dressing material (10) DLP: hemostatic gelatin sponge + cyanoacrylate (10)	NR	Postoperative palatal pain: VAS scale Palatal healing score: measured by comparing the operated palate site to its contralateral counterpart using a visual analog scale (VAS) Willingness to repeat the treatment and painkiller consumption: by questionnaire	Hemostasis was achieved in all patients, regardless method.	DLP: The perception of pain was lower than all other groups (*p* < 0.05) PaG: had less perception of pain compared to the CG (*p* = 0.063) DLP (10%) and PaG (20%): the lower analgesic consumption compared to the CG (50%)	Pain VAS: 1. CG: 2.8; PaG: 1.7; SG: 1.3; PpG: 1.7; DLP: 0.3 2. CG: 2.9; PaG: 1.9; SG: 1.7; PpG: 1.3; DLP: 0.2 3. CG: 2.2; PaG: 1.5; SG: 1.4; PpG: 1.5; DLP: 0.4 4. CG: 1.9; PaG: 1.3; SG: 1.5; PpG: 1.2; DLP: 0.4 5. CG: 1.6; PaG: 1.4; SG: 1.3; PpG: 0.9; DLP: 0.5 6. CG: 1.6; PaG: 1.3; SG: 1.3; PpG: 1.0; DLP: 0.3 7. CG: 1.5; PaG: 1.2; SG: 1.2; PpG: 0.8; DLP: 0.2 10. CG: 1.0; PaG: 0.8; SG: 0.6; PpG: 0.5; DLP: 0.0 14. CG: 1.0; PaG: 0.8; SG: 0.6; PpG: 0.5; DLP: 0.0 Pain (VAS) vs CG (*p* value): PaG: *p* = 0.063 SG: *p* = 0.625 PpG: *p* = 0.328 DLP: *p* = 0.01	The least optimal healing was associated with the CG when compared to the test groups (*P* < 0.001) PaG and DLP: better healing compared to the CG (*P* < 0.001).
Tavelli et al., 2019.	CG: hemostatic sponge (22) TG: hemostatic sponge + cyanoacrylate (22)	NR	Postoperative palatal pain score: VAS scale Willingness to repeat the treatment and painkiller consumption: by questionnaire.	NR	TG had the lowest pain perception at all times (*p* < 0.01) Lower use of analgesics by the TG (10%) compared to the CG (50%) (*p* < 0.01).	The VAS score for TG always lower than 0.6. The peak pain was reached during 3rd day (0.58 ± 0.92) Day 7 (greatest differences CG-TG) – CG 1.8 higher VAS score Day 14 (lowest differences CG-TG) – CG 0.4 higher VAS score	NR
Stavropoulou et al., 2019.	CG: Suture Group (18) TG: cyanoacrylate (17)	CG: 7.31 ± 2.19 TG: 2.16 ± 1.21 Difference between methods (*p* < 0.0001)	Postoperative palatal pain and discomfort score: VAS scale on the first week after surgery Time required for suture placement or cyanoacrylate application The analgesic intake.	NR	NS between the groups for the discomfort and pain level during the first postoperative day and week NS between cyanoacrylate and suture groups for analgesic intake.	Discomfort level (palate) –first week: CG: 1.49 ± 1.96 TG: 1.87 ± 2.25 *p* = 0.56 Pain level (palate) – first day: CG: 1.42 ± 1.88 TG: 1.27 ± 1.92 *p* = 0.96 Pain level (palate) – first week: CG: 1.07 ± 1.87 TG: 1.55 ± 2.32 *p* = 0.28	NS between two methods of wound closure for the modified early-wound healing index (MEHI) (*p* = 0.91)

*NR* Not reported, *NS* Not significance, *FGG* Free Gingival Graft, *NS* Not significant, *CAL* Clinical attachment level, *GR* Gingival recession, *PD* Probing depth, *CoG* Conventional group, *CyG* cyanoacrylato group, *MG* Microsurgery group, *PRF* Platelet-rich fibrin, *BC* butyl-cyanoacrylate, *WG* wet gauze, *IMT* Immediate bleeding time, *DB* Delayed bleeding, *CG* Control group, *PaG* Periacryl group, *SG* Spongostan group, *PpG* Peripac group, *DLP* Double-Layered Protection - Spongostan + Periacryl group, *VAS* Visual Analogic Score, *TG* Test group

The mean year of publication of the included studies was 2015. The studies involved a total of 323 participants. The average number of participants per sample group was 19. The average age of patients was 44.8 years. Regarding gender, the number of female participants was higher. One article did not specify the gender of the patients [2]. The follow-up time for patients ranged from 7 days to 6 months.

Four studies evaluated cyanoacrylate action after free gingival graft surgery in donor areas [2, 9, 13, 14], and only two in the recipient area [1, 10]. The studies were carried out in four countries. Four of the studies were conducted in universities [1, 2, 10, 14] and two in private practices [9, 13]. The surgical procedures in all of these studies were performed by a single calibrated and experienced operator in the field.

### Risk of bias assessment

The different reports from randomized controlled clinical trials demonstrated a low risk of bias for four articles [1, 2, 13, 14]; intermediate for one article [9], in which half of the items were classified as low risk and the other half as unclear risk of bias; and one article presented an unclear risk of bias [10] when the RoB 2.0 tool was applied (Table 4).

**Table 4:** Assessment of the quality and risk of bias of the included studies (RoB 1.0 tool, Cochrane Collaboration Manual)

Authors and year	Selection bias		Performance bias	Detection bias	Attrition bias	Reporting bias
	1. Generation of random sequence	2. Allocation concealment	3. Blinding participants and professionals	4. Blinding evaluators to the outcome	5. Incomplete outcomes	6. Selective outcome report
Barbosa et al. 2009	Unclear/?	Unclear/?	Unclear/?	Unclear/?	Unclear/?	Unclear/?
Gümüş and Buduneli. 2014	Low/+	Low/+	Low/+	Unclear/?	Unclear/?	Low/+
Ozcan et al. 2017	Low/+	Low/+	Low/+	Low/+	Low/+	Low/+
Tavelli et al. 2018	Low/+	Low/+	Low/+	Low/+	Unclear/?	Unclear/?
Tavelli et al. 2019	Low/+	Low/+	Low/+	Unclear/?	Unclear/?	Unclear/?
Stavropoulou et al., 2019.	Low/+	Low/+	Low/+	Unclear/?	Low/+	Low/+

*Low/+* Low risk of bias, *High/−* High risk of bias, *Unclear/?* Uncertain risk of bias

Except for the study of Barbosa et al. (2009) [10], all the other articles described the randomization process and allocation concealment in their methodologies, with randomization using a computerized table [2, 9, 13, 14] or sealed envelope [1], as well as the allocation concealment by a blind investigator and sealed opaque envelopes which were only opened at the time of surgery [1, 2, 9, 13, 14]. The blinding of participants and professionals was reported in four papers [1, 2, 9, 13] and not specified in only one [10]. The blinding of the evaluators was reported in two articles [2, 13] and not explained in four [1, 9, 10, 14].

### Surgical characteristics and effects of cyanoacrylate

Root coverage surgeries were performed in areas with the anterior gingival recession in four studies [1, 2, 9, 10], and in the anterior and posterior regions in two studies [13, 14]. Root coverage surgeries were performed in both arches (maxilla and mandible) in four studies [2, 9, 13, 14], and exclusively on the mandible in two studies [1, 10]. The graft donor area was the same in all studies, the posterior palatal mucosa, involving the region between the maxillary first premolar and the first molar. The test group with isolated cyanoacrylate application in the free gingival graft donor or recipient area was presented in five studies [1, 2, 10, 13, 14], with the exception of one study in which cyanoacrylate was applied together with a hemostatic sponge [9].

The hemostatic, analgesic and/or healing effects of isolated cyanoacrylate or associated with another substance were compared with the effects of isolated products or the following materials: conventional suture thread [1, 10, 13, 14], 7–0 microsurgery sutures [1], sterile gauze pad [2], hemostatic sponge [9, 13] and periodontal surgical cement [13]. The description of the included studies, population characterization (sample size, gender, and mean age), indication for free gingival graft (area/type of recession), sample unit (donor or recipient area of the free gingival graft), type of intervention (graft fixation or wound dressing), and follow up are presented in Table 2. The summary of the analyzed studies with study groups, evaluation time, evaluated parameters, hemostatic and analgesic outcomes, VAS score (Visual Analogic Scale), and healing outcomes are shown in Table 3.

Two studies correlated the application of cyanoacrylate to the surgery duration [1, 14], the first with a statistically significant difference (*p* < 0.05) between the Cyanoacrylate group (26.87 ± 2.13 min) and the conventional suture groups (37.33 ± 2.13 min) and microsurgery (44.13 ± 3.46 min) [1]; and the second with regards to the time required for application of the cyanoacrylate adhesive or suturing, where the mean value for the cyanoacrylate group was 2.16 ± 1.21 min and 7.31 ± 2.19 min for the suture group [14]. The difference between the two methods of wound closure was 5.15 min (*P* < 0.0001).

Another study analyzed immediate hemostasis and bleeding during the first week after the FGG surgery [2]. The authors concluded that the use of cyanoacrylate associated with platelet-rich fibrin (PRF) exhibited shorter bleeding time (0.57 ± 0.15 min) when compared to an isolated cyanoacrylate group (1.65 ± 0.69 min), which in turn had a shorter bleeding time than the bleeding time of the compress group with sterile gauze (3.18 ± 0.61 min). This study also analyzed bleeding during the first week after surgery, in which the following results were found: the PRF group associated with cyanoacrylate did not present bleeding within 7 days after free gingival graft surgery; the isolated cyanoacrylate group showed 82% bleeding on the first day and no bleeding from the 2nd to the 7th day after surgery; while the sterile gauze pad group showed bleeding every day in the first week.

Analgesia was addressed by five studies [1, 2, 9, 13, 14]. The study by Gümüş & Buduneli (2014) [1] observed that pain in the recipient area was lower in the cyanoacrylate group at all follow-up times from the 1st to the 6th post-surgery days (*p* < 0.05) when compared to pain referred to in the other two groups (conventional suture and microsurgery). Ozcan et al. (2017) [2] demonstrated that the PRF group associated with cyanoacrylate obtained superior analgesia results until the 6th day of follow-up; however, from that day on there were no longer statistically significant differences between the PRF group and the isolated cyanoacrylate group. The isolated cyanocrilate group also showed superior analgesia results compared to the sterile gauze dressing group in the first 2 weeks. All groups showed similar pain scores in the postoperative VAS from the 14th day.

For Tavelli et al. (2019) [9], the hemostatic sponge group associated with cyanoacrylate had the lowest perception of pain by the patients at all follow-up times from the 1st to the 14th days (*p* < 0.01), and exhibited the lowest consumption of analgesics (10%) compared to the control group (50%). In other study, Tavelli et al. (2018) [13], showed that the hemostatic sponge group associated with cyanoacrylate showed a lower perception of pain compared to the other groups (*p* < 0.05). The isolated cyanoacrylate group had a numerically lower perception of pain compared to the suture group. The cyanoacrylate associated hemostatic sponge group (10%) and isolated cyanoacrylate (20%) groups showed the lowest analgesic consumption. Stavropoulou et al. (2019) [14] showed that, during the first postoperative week, pain was reported in the palate on the first postoperative day and week, and it also the analgesic intake during the first postoperative week; but the cyanoacrylate and suture groups had not different statistically significant. Healing was reported in five studies [1, 2, 10, 13, 14]. Healing was similar between the tested groups (conventional suture and cyanoacrylate) in the study by Barbosa et al. (2009) [10], with no statistically significant differences found. In the first study [10], the dimensions (height and length) of keratinized gingiva before and after surgery were recorded with a digital caliper after using Schiller’s solution. In the second study [14], the length and height of the wound at the palatal site were measured. The modified early-wound healing index (MEHI) was recorded based on the clinical presentation and the presence of fibrin and necrosis, with classification MEHI 1: complete flap closure without fibrin line at the palate; MEHI 2: complete flap closure with fibrin line at the palate; MEHI 3: complete flap closure with small fibrin clot at the palate; MEHI 4: incomplete flap closure with partial necrosis of the palatal tissue; and MEHI 5: incomplete flap closure with complete necrosis of the palatal tissue (>50% of former flap).

Gümüş & Buduneli (2014) [1] used a specific software (ImageJ, National Institutes of Health, Bethesda, Maryland, USA) to analyze and determine clinical photographs of grafted area, keratinized tissue, and gingival recession. This study observed that the cyanoacrylate group (18.53%) had lesser graft retraction (*p* < 0.05) in their study, meaning better tissue healing than the other two groups (conventional suture-32.50% and microsurgical suture-36.33%) at 6 months follow-up. In the study by Ozcan et al. (2017) [2], showed that the PRF group associated with cyanoacrylate showed had better healing than in the isolated cyanoacrylate group only in the 2nd week of follow-up, and the results regarding healing (complete epithelialization) in the 1st, 3rd and 4th weeks were similar between these two groups. The isolated cyanoacrylate group also exhibited a similar healing effect to that of the sterile gauze dressing group in the 1st and 2nd weeks of follow-up, and higher than the latter in the 3rd week. However, all groups in the 1st and 4th weeks of follow-up had similar healing effects. The wound epithelialization parameter was assessed clinically and by means of color photographs, and only one blinded, experienced examiner performed all clinical measurements.

Finally, Tavelli et al., 2018 [13] used the palatal healing score to visually evaluate the healing of the palatal wound by comparing the operated palate site to its contralateral counterpart using a VAS. The authors observed that the least optimal healing for the palatal surface was associated with the control group when compared to the mean values of the four test groups (*p* < 0.001). In addition, cyanoacrylate bioadhesive group and DLP (Double-layered Protection with cyanoacrylate and hemostatic gelatin sponge) group promoted better healing compared to the control group with suturing only (*p* < 0.001).

## Discussion

The results of the present systematic review indicate that the use of cyanoacrylate associated or not with the hemostatic sponge or the PRF was more effective in hemostasis, healing, and analgesia compared to the control group, however on the basis of relatively limited clinical evidence. Furthermore, groups with the cyanoacrylate in association showed superior effects than the isolated cyanoacrylate group.

Free gingival graft (FGG) is a widely used surgical procedure to increase the dimensions of the inserted gingiva. Its autogenous character, maintenance of tissue keratinization, predictability of the results and the ease of the technique have made FGG considered as the gold standard among gingival augmentation procedures [15, 16]. However, as the FGG produces have two surgical wounds, one in the donor area and the other in the graft recipient area, there is a need for these wounds to be closed with materials which promote hemostasis, analgesia and which can also facilitate the healing process, as recommended in the literature [15–17].

Cyanoacrylate stands out among the materials on the market for the closure of surgical wounds which has been pointed out by different authors [1, 3, 4, 6] as being an alternative to conventional suture materials due to their hemostatic [18, 19], analgesic [9, 18, 19], and healing properties [6, 9, 19]. Moreover, studies [1, 14] demonstrate that the application of cyanoacrylate records a shorter surgery time than the suturing groups (*p* < 0.05). This demonstrates that the use of cyanoacrylate to close the free gingival graft surgical wound can optimize professionals’ and patients’ time. Furthermore, these authors related this shorter operating time to reduced inflammation and consequently edema and pain in the postoperative period.

Cyanoacrylate can be used in several oral surgical procedures with clinical and histological activity, as it has been shown to work as an excellent surgical cement and hemostatic agent which is well tolerated by the tissues, thus facilitating the healing process and also reduces the surgical time [19–21]. The reduction in surgical time influences better healing, since a longer surgical time can influence healing by secondary intention [22]. Moreover, surgical wound healing can also be optimized by an adequate approach of the edges and correct isolation [23], corroborating the results of this systematic review.

Considering that cyanoacrylate seals the edges of the surgical wound acting as a superficial plug without allowing space for fluids or other oral products to interfere during healing, thus isolating the wound margins from the actions of saliva, food debris and biofilm [18], it is possible to suggest that such adhesive material also contributes to better healing and less tissue shrinkage because it has this additional occlusive advantage. This is in agreement with Santos et al. (1990) [24], that revealed that wounds sutured with silk threads showed more intense signs of inflammation and greater tissue contraction than those treated with cyanoacrylate.

In the case of FGG, the tissue contraction usually resulting from the healing process seems to occur in two phases: first, during the formation of a network of blood vessels in the graft, and soon after when the grafts integrate with the recipient area [25]. The graft stabilization in the recipient area using cyanoacrylate results in an atraumatic procedure, with less graft shrinkage being less than by sutures. Factors such as atraumatic surgical technique, thickness, and rapid stabilization of the graft are essential to reduce its shrinkage [1].

Two studies [10, 14] differed in the healing results and showed similar findings that healing was similar between the tested groups (conventional suture and cyanoacrylate). In contrast, most of the studies [1, 2, 13] observed that the cyanoacrylate group had smaller graft retraction (*p* < 0.05) or better epithelialization, meaning better tissue healing than the other test groups. The divergence in results between these studies can be attributed to the different methodologies used, and not only involving the investigated groups, but also the follow-up times and the strategy of evaluation.

The literature [3, 19, 23] points out that the use of cyanoacrylate to close intraoral wounds seems promising, not only due to its occlusive and healing properties, but also for hemostatic and analgesic properties. When investigating the application of cyanocrilate as an alternative to suture intraoral and extra-oral wounds, somestudies [6, 21, 26] concluded that cyanoacrylate is faster, more reliable, less painful and causes better hemostasis than conventional suturing.

The hemostatic potential of cyanoacrylate in preventing bleeding, associated or not with coagulation disorders in oral surgery, has been previously evaluated [18, 27–30]. These studies demonstrate that local hemostasis was obtained immediately when cyanoacrylate was used. Though, the mechanism by which cyanoacrylate promotes hemostasis is not clear. One hypothesis is that the cyanoacrylate ester forms a macrofilm causing mechanical blockage to slow blood flow, providing a surface agent to activate the clotting cascade [29]. There is evidence that the film forms a porous mass that is invaded with blood with subsequent clotting within the pores of the adhesive [29]. The only study included in the review regarding hemostasia concluded that cyanoacrylate alone or associated with PRF has superior hemostatic action to that of sterile wet gauze compression [2].

Cyanoacrylate was widely accepted by patients when used as a protector for the graft donor region due to pain relief and reduced discomfort during feeding [19]. The ability of the cyanoacrylate to form a protective layer that isolates the wound in the oral cavity is the main reason responsible for the decrease in postoperative pain [1, 2, 9, 13]. Thus, the lowest analgesic consumption which occurred when using cyanoacrylate was probably due to less pain experienced when compared with suturing [9, 13].

Cyanoacrylate has been used in several oral surgical procedures, including surgeries for FGG, in which it has been shown to be comparable or even superior to suturing due to its greater ease of use, reduced operative time, immediate hemostasis production and for promoting postoperative comfort [1, 2, 9, 10, 13, 18]. However, additional studies involving a larger sample size, methodological standardization and longer follow-up are necessary to demonstrate such effects in order to more clearly show the effectiveness of cyanoacrylate in closing surgical wounds from FGG and its hemostatic, analgesic and healing effects.

The included articles were generally considered to have a low risk of bias, as there was no high risk of bias classification in any of the items assessed in the articles included in this review. The items considered “unclear risk of bias” fell into this category when the parameter was reported, but the precise execution was not clear. As an example, the item “Generation of the random sequence” was classified as unclear risk of bias in the article by Barbosa et al. (2009) [10] because, even though their article presented the information that the study had been randomized, the necessary information for correctly judging the methodology for executing the randomization process was insufficient.

Heterogeneity in the free gingival graft recipient area was observed in the included studies; however, the graft donor area was the same in all studies, namely: the posterior palatal mucosa, usually involving the region between the first premolar and the maxillary first molar. Only one study evaluated the effect of applying or not applying cyanoacrylate on a hemostatic sponge, but it did not have an exclusive cyanoacrylate group. In addition, this review did not assess the effect on clinical the increase in keratinized tissue and includes studies which use cyanoacrylate for different indications, thus making comparisons difficult. Given the heterogeneity of the outcomes, no Meta-analysis was performed.

## Conclusion

The cyanoacrylate-based adhesives (either exclusive or associated) generated; less perception and experience of pain, reduced postoperative discomfort and lowered analgesic consumption; did not interfere in the healing process, presented lower graft shrinkage, and promoted a better epithelialization response compared with suturing. In addition, cyanoacrylate promotes reduced surgical time, including reducing the number of follow-up visits as suture removal becomes unnecessary. These characteristics of cyanoacrylate suggest its exclusive use or associated with other techniques and substances as an alternative to conventional suturing in free gingival graft surgeries in both the donor and recipient areas. The hemostatic activity could not be confirmed because only one study presented this information. However, relative methodological limitations of the selected studies and the total number of study subjects (*n* = 323) suggest considerable caution when interpreting the results and highlight the need for more properly designed clinical trials.
